# Development and Evaluation of a Lyophilized Plasma-Based Internal Quality Control for Human Immunodeficiency Virus Rapid Diagnostic Tests

**DOI:** 10.3390/diagnostics16040608

**Published:** 2026-02-19

**Authors:** Siriphailin Jomjunyoung, Wanvisa Treebuphachatsakul, Supaporn Suparak, Nam K. Tran, Gerald J. Kost, Napaporn Apiratmateekul

**Affiliations:** 1 Reference Materials and Innovation Research Unit for Medical Laboratory, Faculty of Allied Health Sciences, Naresuan University, Phitsanulok 65000, Thailand; siriphailinj65@nu.ac.th (S.J.); wanvisab@nu.ac.th (W.T.); 2 Department of Medical Sciences, Ministry of Public Health, Nonthaburi 11000, Thailand; supaporn.su@dmsc.mail.go.th; 3 Department of Medical Technology, Faculty of Allied Health Sciences, Naresuan University, Phitsanulok 65000, Thailand; 4 Computational Pathology and AI Center of Excellence (CPACE), School of Medicine, University of Pittsburgh, Pittsburgh, PA 15213, USA; trannk@upmc.edu; 5 Pathology and Laboratory Medicine, School of Medicine, University of California, Davis, CA 95616, USA

**Keywords:** internal control samples, HIV rapid diagnostic tests, stability study, point-of-care testing

## Abstract

**Background/Objectives:** Rapid diagnostic tests (RDTs) for human immunodeficiency virus (HIV) are widely used, but most kits lack standardized internal quality control (IQC) materials. In this study, we aimed to develop and evaluate a plasma-based IQC compatible with five HIV RDT brands and with proven long-term stability. **Methods:** Control samples at three reactivity levels were tested with five HIV RDT kits in lyophilized and liquid forms. Lyophilized samples were produced with and without trehalose, whereas liquid samples were prepared with and without StabilZyme™ SELECT Stabilizer (Stabilizer). Accelerated stability testing was performed at 37 °C and 45 °C for 28 days, and the most stable formulation was selected for long-term storage at 4 ± 2 °C and 25 ± 5 °C. Stability was assessed based on test-line visibility and signal intensity. Signal-intensity trends were analyzed using simple linear regression with a *t*-test on the slope; samples were considered stable when no significant trend was detected (*p* > 0.05). **Results:** Reactivity measured using the Elecsys HIV combi PT assay yielded cutoff index (COI) values of 772.65 (1:8) for the strong-positive control and 269.95 (1:25) for the weak-positive control. Trehalose-containing lyophilized samples maintained reactivity under accelerated testing at 37 and 45 °C and for 6 months at 4 ± 2 °C and 25 ± 5 °C, with no significant change in signal intensity (*p* > 0.05). **Conclusions:** The plasma-based IQC materials were compatible with all five HIV RDTs, and trehalose-stabilized lyophilized plasma showed high stability, supporting transport and storage without strict cold-chain requirements.

## 1. Introduction

According to the World Health Organization, human immunodeficiency virus (HIV) diagnosis requires at least three sequentially reactive results obtained using different test kits to enhance diagnostic accuracy and reduce the likelihood of false-positive results [1,2,3]. Similarly, Thailand’s national guidelines recommend either three distinct serological assays or a two-step protocol with an initial screening test followed by a confirmatory assay [4].

Data from the National Institute of Health Science Research, Department of Medical Sciences, Ministry of Public Health, indicate that RDTs are widely adopted in routine clinical practice. Most of the 261 laboratories participating in the External Quality Assessment program across nine provinces in central Thailand reported incorporating one to two HIV RDT kits into their diagnostic workflows [unpublished data] [5]. These observations underscore the integral role of RDTs in HIV screening across various levels of healthcare services. Despite their widespread adoption, most commercially available RDT kits lack internal quality control (IQC) materials, an essential component for ensuring analytical reliability, verifying test validity, detecting procedural errors, and maintaining consistent performance during routine testing. Recognizing the importance of this process, the International Organization of Standards (ISO) 15189:2022 explicitly recommends implementing IQC, as it is a crucial measure for ensuring the reliability of medical laboratory results, thereby reinforcing the need for robust quality control even in rapid diagnostic testing contexts [6].

Previous studies have investigated the use of dried tube specimens (DTSs), prepared from HIV-positive plasma, as potential IQC materials. These studies demonstrated that DTS offers superior stability compared with liquid controls. DTS samples can be stored at 4 and 25 °C for at least 4 weeks and exhibit minimal degradation even at elevated temperatures of 37 and 45 °C [7]. Long-term stability has also been confirmed; for instance, HIV-DTS maintained 100% sensitivity and specificity after 1 year at 2–8 °C, supporting their suitability as effective IQC materials [8].

Trehalose, a disaccharide with cryoprotective and stabilizing properties, has been used to enhance protein preservation during lyophilization [9]. Its inclusion extended storage duration without compromising antigen reactivity, making it particularly beneficial for quality control and proficiency testing in resource-limited settings. Lyophilization, comprising pre-freezing, primary drying, and secondary drying, is a widely used technique for preserving biological materials. Precise control of the primary drying endpoint is critical to ensure product integrity and batch-to-batch consistency [10].

This study aimed to assess the usability of three levels of IQC samples, strong positive, weak positive, and negative, for use in five rapid HIV tests widely applied in laboratories participating in the external quality assessment (EQA) program across nine provinces in central Thailand. Evaluations focused on determining sample homogeneity and examining the utility of a stabilizing agent to prolong plasma shelf life for IQC purposes. Furthermore, the prepared samples were evaluated for stability under both accelerated and long-term storage conditions, including refrigerated and ambient environments [11,12].

## 2. Materials and Methods

This study was designed to evaluate which IQC formulation provides superior stability by comparing lyophilized samples with and without trehalose to liquid IQC samples with and without stabilizer, using five HIV rapid diagnostic test kits and cutoff (COI) measurements from the Elecsys HIV combi PT assay (Roche Diagnostics GmbH, Rotkreuz, Switzerland). In addition, we examined whether changes in COI values over time and across storage conditions remained statistically non-significant, using independent *t*-tests and regression analysis (non-significant *t*-tests and regression slopes not different from zero; *p* > 0.05), in accordance with the stability criteria specified in ISO 33405:2024 [13]. Furthermore, the IQC samples were required to be homogeneous, and for the lyophilized IQC, we also evaluated the duration for which the reconstituted samples remained stable.

### 2.1. Plasma Source and HIV Testing

HIV-negative fresh frozen plasma (FFP) samples were collected from donor units confirmed as non-reactive through routine HIV screening. All FFP samples were sourced from donors screened by the Thai Red Cross using both serological and nucleic acid testing methods. Before analysis, all specimens were anonymized to ensure donor confidentiality. This study was approved by the Institutional Review Boards of Naresuan University (COA No. 118/2023; IRB No.P1-0024/2566) and the Thai Red Cross Society (COA No. NBC 4/2024).

HIV screening was performed using the Alinity i HIV Ag/Ab Combo (Abbott Diagnostics, Chicago, IL, USA) and the Elecsys HIV combi PT. Reactive samples were confirmed using the Geenius™ HIV 1/2 Confirmatory Assay (Bio-Rad Laboratories, Hercules, CA, USA).

A total of four plasma specimens (three HIV-positive and one HIV-negative) were selected for subsequent analysis. The three HIV-positive specimens were pooled to produce a composite positive control, which was validated using the Geenius™ HIV 1/2 Confirmatory Assay. The confirmatory results demonstrated a complete HIV-1 band profile, ensuring the suitability of the pooled material for IQC preparation.

### 2.2. Interpretation of HIV Rapid Test Results

Five commercially available HIV RDT kits were evaluated and anonymized as Kit A–E: Determine™ HIV-1/2 (Abbott Diagnostic Medical, Chiba, Japan; Kit A), STANDARD Q HIV 1/2 Ab 3-Line (SD Biosensor Inc., Suwon, South Korea; Kit B), Diagnostic Kit for HIV (1+2) A V2 (Livzon, Zhuhai, China; Kit C), Wondfo One Step HIV 1/2 (Wondfo, Guangzhou, China; Kit D), and One Step Anti-HIV (1&2) Tri-Line (InTec Products, Inc., Xiamen, China; Kit E). All tests were performed according to the manufacturer’s instructions and interpreted within 15–20 min.

**Figure 1:**
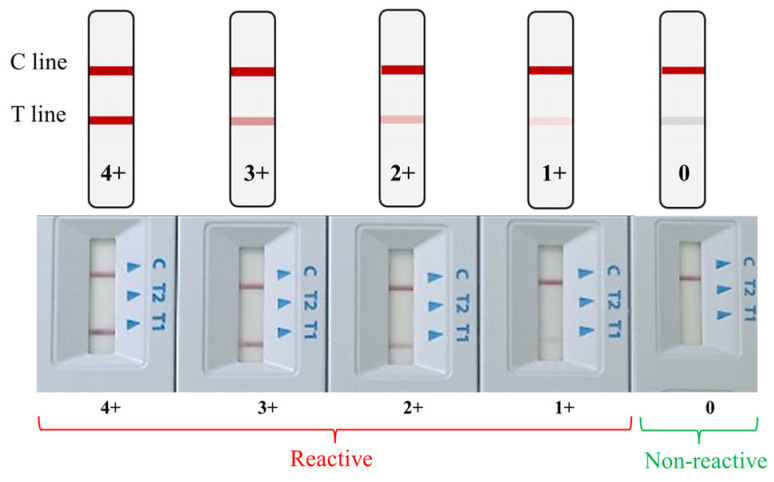
Test line intensity represents the reactivity level (Reactive 4+ to 1+, Non-reactive).

A result was considered positive when both the control (C) and test (T) lines were visible, and negative when only the C line appeared. Tests lacking a C line were classified as invalid and repeated. To ensure consistency, all results were independently interpreted by two blinded personnel. Reactivity was graded ordinally from 4+ to 1+ based on the intensity of the T line relative to the C line. Faint or barely visible T lines were interpreted as non-reactive. The C line served as the internal validity control (Figure 1).

### 2.3. Specimen Collection and Selection

Four plasma specimens were selected and confirmed using reference assays, comprising three HIV-positive and one HIV-negative FFP samples. The HIV-positive samples showed varying reactivity on the Alinity i HIV Ag/Ab Combo and Elecsys HIV combi PT assays: FFP1 (S/Co = 735.00, COI = 142.20), FFP2 (S/Co = 310.23, COI = 753.00), and FFP3 (S/Co = 205.27, COI = 1575.00). The HIV-negative specimen was non-reactive (S/Co = 0.29, COI = 0.25). The three HIV-positive samples were pooled to generate a composite positive control, yielding a final COI of 1808.00. The pooled sample was validated using a confirmatory assay. It demonstrated a complete HIV-1 band profile, including p31, gp160, p24, and gp41, confirming the presence of a broad polyclonal antibody response against major viral structural proteins (Env, Gag, and Pol products). This comprehensive profile ensures that the pooled material is representative of local circulating strains and remains robust despite potential viral genomic drift, with signal intensities stronger than those of any individual specimen. This pooled material was subsequently used for IQC preparation.

### 2.4. Determination of Optimal Concentration

Serial dilutions of HIV-positive plasma were prepared in HIV-negative plasma at ratios ranging from 1:2 to 1:1024. Each dilution was tested in triplicate using all five RDTs. In parallel, all dilutions were quantified using the Elecsys HIV combi PT assay to obtain COI values. Visual reactivity was graded from 4+ (strongest) to 1+ (weakest). Based on COI values and corresponding line intensities, strong-positive and weak-positive levels were selected, whereas HIV-negative plasma served as the negative control.

### 2.5. Preparation of the IQC Samples

IQC samples were prepared by pooling HIV-positive plasma for positive controls and HIV-negative plasma for negative controls. Both liquid and lyophilized formulations were produced. Lyophilized samples were prepared with and without trehalose. Liquid samples were prepared with and without StabilZyme™ SELECT Stabilizer (Surmodics, Eden Prairie, MN, USA), a protein-stabilizing reagent used to preserve antibody integrity in lateral-flow assays. For clarity, this reagent is hereafter referred to as “stabilizer.” HIV-negative plasma served as the diluent. Strong-positive (1:8) and weak-positive (1:25) IQC samples were prepared in both formats. All preparations were aliquoted into 0.75-mL vials and stored at –20 °C as Day-0 reference material. Before use in stability studies, each formulation underwent homogeneity assessment to confirm consistency across aliquots. All IQC samples were then tested in triplicate using the five HIV RDT kits, and their semi-quantitative reactivity was verified with the Elecsys HIV combi PT assay. Samples stored at −20 °C served as the reference condition for stability comparisons.

### 2.6. Homogeneity Testing

Ten aliquots from each IQC lot were randomly selected and tested using all five RDT kits under identical conditions. Homogeneity was acceptable when qualitative results were fully concordant. Additionally, aliquots of the lyophilized formulation prepared with trehalose (the most challenging matrix) were tested in duplicate using the Elecsys assay. Inter- and intra-vial variances were statistically evaluated using Cochran’s C test and one-way ANOVA, following ISO 33405:2024 [13,14].

### 2.7. Accelerated Stability Testing

Accelerated stability was assessed by incubating four sets of IQC samples at 37 and 45 °C for 28 days. At predefined intervals (Days 7, 14, 21, and 28), one set was removed and stored at –20 °C until analysis. All samples were tested using the five HIV RDT kits and the Elecsys HIV combi PT assay. Results at each time point were compared with baseline (Day 0) measurements to evaluate potential degradation or loss of reactivity. Different IQC formulations were evaluated in parallel. COI values from stored samples were compared with Day 0 values using independent *t*-tests, in accordance with ISO 33405:2024. In addition, signal-intensity trends were assessed using simple linear regression with a *t*-test applied to the slope to determine whether a significant change occurred during incubation. The formulation showing no qualitative changes, consistent test-line scores, no significant differences in COI values compared with Day 0, and a non-significant regression slope (*p* > 0.05) at both temperatures was defined as the most stable formulation and selected for long-term monitoring [13].

### 2.8. Long-Term Stability Monitoring

Long-term stability was evaluated by storing IQC samples at 4 ± 2 °C and 25 ± 5 °C for 6 months. Aliquots were tested monthly using all five RDT kits. When no qualitative changes were observed, the aliquots were returned to –20 °C and subsequently analyzed using the Elecsys assay. COI values were reported as the mean ± SD. Comparisons between storage conditions were performed using independent *t*-tests, in accordance with ISO 33405:2024.

### 2.9. Reconstitution and Stability Monitoring of Lyophilized IQC Samples

Lyophilized IQC samples were reconstituted with 0.75 mL sterile PBS-Tween (pH 7.4, 50 mM, 0.05% *v*/*v* Tween-20). Each vial was vortexed gently for 10–15 s to ensure complete dissolution and equilibrated at 4 ± 2 °C for 30 min prior to testing. Weak-positive IQC samples were used for reconstitution stability studies. Stability was assessed based on visual intensity and COI values (mean ± SD from replicate measurements). Any decline in test-line intensity or COI from Day 0 was documented.

### 2.10. Statistical Analysis

Temporal stability was evaluated using linear regression analysis of COI values (y) against storage time in days (x). For each IQC formulation and temperature condition, a simple linear regression model (y = b_0_ + b_1_x) was fitted. The regression slope (b_1_) represented the rate of change in COI over time. The standard error of the slope (sb_1_) was calculated from the residual variance, and the statistical significance of the slope was assessed using a two-tailed *t*-test defined as t = |b_1_/sb_1_| with degrees of freedom (df = *n* − 2). A non-significant slope (*p* > 0.05) indicated no meaningful decline in COI over time, and the IQC material was therefore interpreted as sufficiently stable under the tested storage condition. Conversely, a statistically significant negative slope (*p* < 0.05) was interpreted as evidence of degradation. All calculations, including regression coefficients, standard error of slope, t-statistics, and *p*-values, were performed using Microsoft Excel with manually validated formulas consistent with ISO 33405:2024 [13].

## 3. Results

### 3.1. Results of the Optimal Concentration Determination

Serial dilutions of HIV-positive plasma revealed varying detection limits among the five RDT kits. Kit A demonstrated the highest sensitivity (detectable at ~1:1024), followed by Kits B and C (~1:512), Kit D (~1:256), and Kit E (~1:128). All tests were performed in triplicate and yielded consistent results. A summary of the results is presented in Table 1.

Two representative dilutions were selected for IQC preparation. The strong-positive sample at 1:8 showed strong reactivity (3+ to 4+) across all five kits (COI = 772.65). The weak-positive sample at 1:25 showed moderate reactivity (2+ to 3+) with a COI of 269.95.

### 3.2. IQC Sample Preparation Outcomes

All IQC formulations demonstrated the expected reactivity patterns across the five HIV RDT kits. Strong-positive samples (1:8) generated clear and consistent test-line signals in all kits, whereas weak-positive samples (1:25) produced lower but still detectable reactivity, with minor variations in signal intensity depending on the assay. Negative controls showed no visible test lines in any formulation. All IQC samples were tested in triplicate across the five HIV RDT kits, and their semi-quantitative reactivity was further verified using the Elecsys HIV combi PT assay. A summary of the reactivity patterns and COI values is presented in Table 2.

### 3.3. Homogeneity Test Results

Ten vials were randomly selected from each batch and tested using all five HIV RDT kits. All vials demonstrated concordant qualitative reactivity with no observable variation in test-line intensity. Lyophilized trehalose-based IQC underwent additional statistical evaluation using Cochran’s C test and one-way ANOVA. The results confirmed that inter-vial variance remained within acceptable limits for both the strong-positive (C_exp = 0.454 < C_crit = 0.602) and weak-positive (C_exp = 0.428 < C_crit = 0.602) levels. One-way ANOVA further demonstrated no significant between-vial differences, with F-values below the corresponding critical thresholds for the strong-positive (F_cal = 2.426 < F_crit = 3.020) and weak-positive (F_cal = 1.914 < F_crit = 3.020) samples. Together, these findings confirm acceptable inter-vial homogeneity in accordance with ISO 33405:2024 [13,14].

### 3.4. Accelerated Stability Evaluation

**Figure 2:**
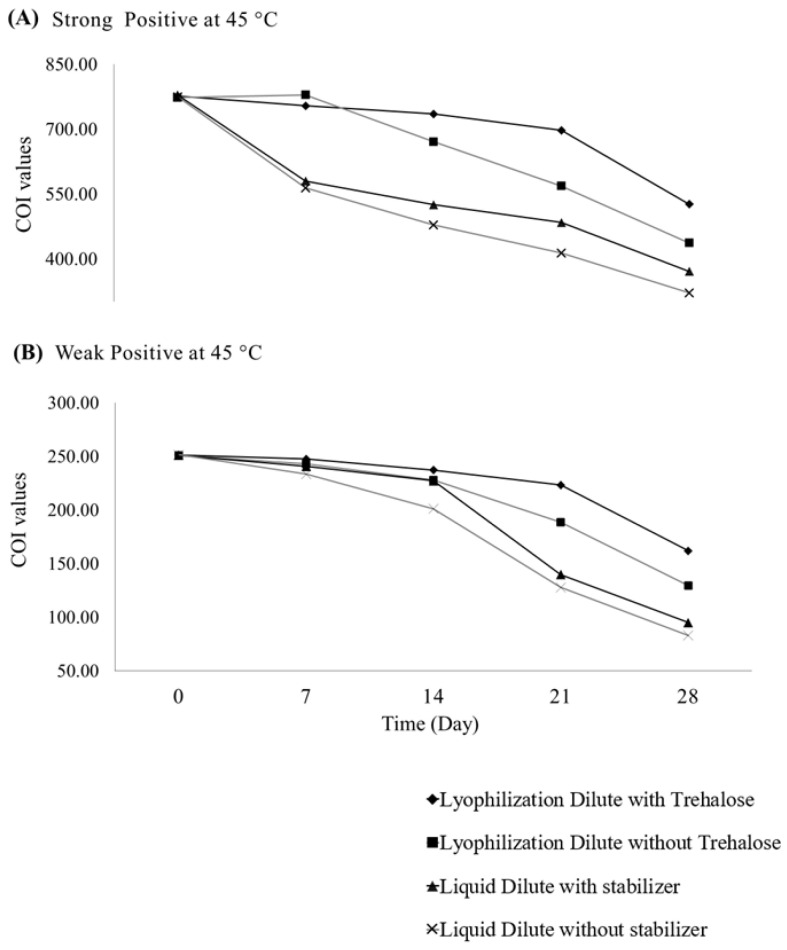
Accelerated stability profiles of strong-positive (**A**) and weak-positive (**B**) IQC samples stored at 45 °C for 28 days. The upper curve represents lyophilized IQC samples containing trehalose, which demonstrated stable performance and showed no statistically significant differences (*p* > 0.05) at either the strong-positive or weak-positive IQC levels. In contrast, the three lower curves correspond to unstable IQC formulations.

Lyophilized IQC samples containing trehalose showed the highest thermal stability at both the strong-positive and weak-positive levels. When stored at 45 °C, these samples exhibited the slowest decline in COI values over 28 days (Figure 2). At all time points, COI values remained comparable to Day 0 (independent *t*-test, *p* > 0.05), and all five HIV RDT kits continued to generate clear and consistent positive reactivity. Linear regression analysis supported these results, with slopes not significantly different from zero (*p* > 0.05), indicating no meaningful degradation under accelerated conditions. In contrast, other formulations—both liquid and lyophilized without trehalose—showed more rapid signal loss, with significant decreases in COI values and visible reductions in test-line intensity (Table 3; Figure 2). Semi-quantitative measurements using the Elecsys HIV combi PT assay confirmed these differences. COI values after storage did not differ significantly from Day 0 for either the strong-positive (t_cal = 3.07 < t_crit = 3.18; *p* = 0.54) or weak-positive (t_cal = 3.12 < t_crit = 3.18; *p* = 0.52) levels, consistent with ISO 33405:2024 [13]. These statistical results confirm that the trehalose-based lyophilized formulation provides superior stability at 45 °C.

### 3.5. Long-Term Stability Monitoring Results

IQC samples stored at 4 ± 2 °C and 25 ± 5 °C remained qualitatively stable for up to 6 months when tested with the five HIV RDT kits. No loss of reactivity was detected under refrigerated storage (4 ± 2 °C). At 25 ± 5 °C, a gradual decline in test-line intensity was observed in Kit B and Kit E after Month 5, whereas Kits A, C, and D maintained stable visual reactivity throughout the study period. No false-negative results occurred under either condition. Quantitative assessment using the Elecsys HIV combi PT assay showed consistent COI values across all months, reported as the mean ± SD from three replicate measurements. Independent *t*-tests comparing COI values between the two storage temperatures showed no statistically significant differences (*p* > 0.05) for either the strong-positive or weak-positive IQC levels, indicating acceptable long-term stability. The detailed results are summarized in Table 4.

### 3.6. Reconstitution and Post-Reconstitution Stability Result

Reconstituted lyophilized weak-positive IQC samples showed stable reactivity across all five HIV RDT kits on Day 0, confirming complete dissolution and homogeneity. After storage at 4 ± 2 °C for 6 months, the reconstituted samples continued to yield reactive results across all five kits, with no noticeable reduction in test-line intensity. COI values obtained from the Elecsys HIV combi PT assay also remained stable. The mean (SD) COI was 278.80 (2.91) at Day 0 and 271.30 (2.55) at 6 months, indicating no meaningful loss of signal during post-reconstitution storage.

## 4. Discussion

The findings of this study show that trehalose-based lyophilized plasma provides a highly reliable IQC formulation, demonstrating strong stability, homogeneity, and consistent reactivity across five HIV RDT kits and a quantitative immunoassay. The IQC samples maintained stable COI values under both accelerated and long-term storage, confirming their suitability for routine HIV rapid testing. Trehalose effectively protected antigenic components during lyophilization, allowing samples to remain reactive for 28 days at 37 and 45 °C—temperatures representative of extreme transport and field conditions in tropical regions. These results align with previous reports on the protective effect of trehalose at elevated temperatures [9]. The superior stability of trehalose-based lyophilized IQC observed in this study can be attributed to two primary mechanisms: the water replacement hypothesis and vitrification [15]. According to the water replacement hypothesis, trehalose molecules form hydrogen bonds with the polar residues of plasma proteins (e.g., immunoglobulins) as water is removed during lyophilization. This interaction effectively substitutes the water shell that normally stabilizes the protein structure, thereby preventing denaturation and aggregation [16]. Concurrently, the vitrification hypothesis suggests that trehalose forms a high-viscosity, non-crystalline glassy matrix (high glass transition temperature) upon drying. This glassy state restricts the molecular mobility of the encapsulated proteins, significantly slowing down degradation kinetics even under thermal stress conditions such as those mimicked in our accelerated stability testing at 45 °C [17]. These properties make trehalose an ideal excipient for preserving antibody reactivity in tropical climates. The robust performance at 45 °C is particularly relevant for Thailand, where ambient temperatures can reach 44 °C, supporting its use in IQC formulations for high-temperature settings [18]. Our findings directly address the gap highlighted in the introduction, namely, the widespread use of HIV RDTs in settings where appropriate IQC materials are often unavailable. We show that locally produced, trehalose-stabilized lyophilized IQC samples remain stable under both refrigerated and ambient storage and perform consistently across five different HIV RDT kits. By providing a feasible and affordable source of standardized IQC material, this work offers a practical pathway for implementing the ISO 15189:2022 recommendation for routine IQC in laboratories that rely on RDT-based HIV screening [6]. Adoption of such controls may strengthen analytical reliability, improve detection of procedural errors, and ultimately reduce the risk of false-negative or false-positive HIV results that adversely affect patient counselling, timely ART initiation, and public health decision-making. Beyond use in individual laboratories, these locally manufactured IQC materials could be integrated into national external quality assessment programs or regional HIV testing networks, providing a scalable approach to strengthening quality assurance for rapid tests in other resource-limited settings. In terms of economic viability, the in-house production of this IQC material offers a significant cost advantage over commercially available counterparts. Specifically, the estimated unit cost of the in-house preparation represents a 50% reduction compared to imported products. This substantial saving is primarily attributed to the utilization of locally sourced raw materials and the elimination of international logistics costs and import duties. Regarding routine implementation, IQC samples should be analyzed on a daily basis, or with each new batch or lot of test kits, to ensure consistent analytical performance.

Overall, the developed IQC materials were suitable for routine use. Homogeneity testing showed acceptable vial-to-vial consistency, with Cochran’s C and one-way ANOVA revealing no excessive variance or significant between-vial differences, in line with ISO 33405:2024 [13]. Long-term stability testing demonstrated that samples stored at 4 ± 2 °C remained fully reactive for at least 6 months, while those stored at 25 ± 5 °C showed reduced band intensity in two kits after Month 5, consistent with known antigen variability among commercial RDTs. In addition, results from Table 4 suggest that 4 ± 2 °C is the optimal storage condition due to the consistently stable COI values observed. However, storage at 25 ± 5 °C still preserved acceptable reactivity, supporting use in settings without refrigeration.

A technical review of lateral flow immunoassays emphasizes that test performance depends primarily on the affinity and specificity of the antibodies or antigens, as well as the quality of the conjugate pad and immobilization process. These factors directly influence the consistency and intensity of color development on the test strip [19,20]. The variability in antigen composition among kits (one to three antigens) likely explains the differences in optimal dilution and test-line intensity. This highlights the need for kit-specific considerations when establishing IQC dilution schemes. The kits contained one to three HIV-1 antigens, which contributed to differences in test-line intensity and analytical sensitivity. As a result, future IQC protocols may need two dilution schemes: one for kits with lower antigen complexity and another for kits incorporating three antigens.

Simple linear regression of COI values over time was chosen to detect temporal trends in stability, in line with ISO 33405:2024 recommendations. This approach directly evaluates degradation by testing whether the regression slope differs significantly from zero, rather than comparing mean COI values at each time point. All analyses followed the ISO 33405 framework, which does not mandate specialized statistical software [13].

This study has two main limitations. First, only HIV-1-positive plasma was assessed; inclusion of HIV-2-positive specimens is needed to improve generalizability. Second, only five RDT kits were evaluated, which does not fully represent all assays available in Thailand. Future work will include a broader range of commercially available test kits.

In conclusion, this study demonstrates the feasibility of preparing and implementing three levels of IQC samples—strong positive, weak positive, and negative—for use with HIV rapid diagnostic tests. The incorporation of stabilizing agents, particularly trehalose, effectively preserved lyophilized plasma, which showed good stability under both accelerated and long-term evaluations. The IQC samples maintained homogeneity and consistent reactivity for at least 6 months under refrigerated (4 ± 2 °C) and ambient (25 ± 5 °C) storage conditions, supporting their suitability for routine quality control in resource-limited settings. These findings provide a practical foundation for developing stable IQC materials that can enhance quality assurance in HIV testing programs.

## Figures and Tables

**Table 1:** Reaction intensity across serial dilutions of human immunodeficiency virus (HIV)-positive plasma using five HIV rapid diagnostic test kits.

Reaction Intensity Level of Rapid Tests										
Sample	Kit-A		Kit-B		Kit-C		Kit-D		Kit-E	
	LQ	LP	LQ	LP	LQ	LP	LQ	LP	LQ	LP
Stock-pool	4+	4+	4+	4+	4+	4+	4+	4+	3+	3+
Pool-1:2	4+	4+	4+	4+	4+	4+	4+	4+	3+	3+
Pool-1:4	4+	4+	4+	4+	4+	4+	4+	4+	3+	3+
Pool-1:8	4+	4+	4+	4+	4+	4+	4+	4+	3+	3+
Pool-1:16	4+	4+	4+	4+	4+	4+	3+	3+	2+	2+
Pool-1:25	3+	3+	3+	3+	3+	3+	3+	3+	2+	2+
Pool-1:32	3+	3+	3+	3+	3+	3+	3+	3+	1+	1+
Pool-1:64	3+	3+	2+	2+	2+	2+	2+	2+	1+	1+
Pool-1:128	2+	3+	1+	1+	1+	1+	1+	1+	0	0
Pool-1:256	1+	2+	1+	1+	1+	1+	0	0	0	0
Pool-1:512	1+	1+	0	0	0	0	0	0	0	0
Pool-1:1014	0	0	0	0	0	0	0	0	0	0

LQ: liquid plasma; LP: lyophilized plasma. Reaction intensity was visually scored on a 0 to 4+ scale, where 0 indicates a non-reactive result, and 4+ indicates the strongest visible test line. All tests were performed in triplicate and yielded consistent results.

**Table 2:** Summary of HIV rapid test reactivity and COI values for IQC samples prepared with different diluents.

Levels of IQC Sample	Test Kits	IQC Liquid		IQC Lyophilization	
		Dilute with Stabilizer	Dilute Without Stabilizer	Dilute with Trehalose	Dilute Without Trehalose
Strong positive (Dilution 1:8)	Kit-A, B, C, D	4+	4+	4+	4+
	Kit-E	3+	3+	3+	3+
	Elecsys HIV combi PT	781.10	776.00	774.50	772.65
Weak positive (Dilution 1:25)	Kit-A, B, C, D	3+	3+	3+	3+
	Kit-E	2+	2+	2+	2+
	Elecsys HIV combi PT	280.80	275.35	269.95	261.10
Negative	All five rapid HIV kits	-	-	-	-
	Elecsys HIV combi PT	0.286	0.261	0.379	0.328

IQC status was assessed using HIV rapid antibody tests, where “+” indicates a reactive result and “-” indicates a non-reactive result. COI refers to the sample-to-cutoff index derived from the Elecsys HIV combi PT assay. A COI value ≥ 1.0 was considered reactive.

**Table 3:** Visual reactivity of IQC samples stored at 37 °C and 45 °C for 28 days using different stabilizing agents across five HIV rapid test kits.

Test Kits	IQC Sample	Temp (°C)	Reaction Intensity Level of Rapid HIV Antibody Tests									
			Strong Positive (Day)					Weak Positive (Day)				
			0	7	14	21	28	0	7	14	21	28
KIT A, B, C, D	Lyophilization dilute with trehalose	37 °C	4+	4+	4+	4+	4+	3+	3+	3+	3+	3+
		45 °C	4+	4+	4+	4+	4+	3+	3+	3+	3+	3+
	Lyophilization dilute without trehalose	37 °C	4+	4+	4+	4+	3+	3+	3+	3+	2+	2+
		45 °C	4+	4+	4+	3+	3+	3+	3+	3+	2+	2+
	Liquid dilute with stabilizer	37 °C	4+	4+	4+	4+	4+	3+	3+	3+	3+	3+
		45 °C	4+	4+	4+	4+	3+	3+	3+	3+	3+	2+
	Liquid dilute without stabilizer	37 °C	4+	4+	4+	3+	2+	3+	3+	3+	2+	2+
		45 °C	4+	4+	3+	3+	2+	3+	3+	3+	2+	2+
KIT-E	Lyophilization dilute with trehalose	37 °C	3+	3+	3+	3+	3+	2+	2+	2+	2+	2+
		45 °C	3+	3+	3+	3+	3+	2+	2+	2+	2+	2+
	Lyophilization dilute without trehalose	37 °C	3+	3+	3+	3+	2+	2+	2+	2+	2+	1+
		45 °C	3+	3+	3+	2+	2+	2+	2+	2+	1+	1+
	Liquid dilute with stabilizer	37 °C	3+	3+	3+	3+	3+	2+	2+	2+	2+	2+
		45 °C	3+	3+	3+	3+	2+	2+	2+	2+	2+	1+
	Liquid dilute without stabilizer	37 °C	3+	3+	3+	3+	2+	2+	2+	2+	2+	1+
		45 °C	3+	3+	3+	2+	1+	2+	2+	2+	1+	1+

Visual reactivity of strong-positive and weak-positive IQC samples stored at 37 and 45 °C for 28 days, evaluated using five commercial HIV rapid test kits. Samples were prepared using four formulations (lyophilized with trehalose, lyophilized without trehalose, liquid with stabilizer, liquid without stabilizer). Reaction intensity was recorded using an ordinal grading scale from 1+ to 4+, with higher values indicating stronger test-line visibility. “+” denotes reactive HIV antibody results.

**Table 4:** Long-term stability of IQC samples stored at 4 ± 2 °C and 25 ± 5 °C over 6 months, expressed as COI mean (SD) and evaluated using independent *t*-tests.

QC	Temp	COI (Mean, SD)							*t*-Test		
		Day 0	Month 1	Month 2	Month 3	Month 4	Month 5	Month 6	t _cal < t _Crit	*p*-Value	Interpretation
Strong positive	4 ± 2 °C	769.50 (4.47)	765.90 (3.91)	768.60 (5.08)	767.00 (5.09)	767.93 (5.51)	768.33 (3.09)	765.23 (4.82)	1.07 < 2.57	0.36	Acceptable
	25 ± 5 °C	769.50 (4.47)	766.82 (2.37)	767.42 (1.77)	766.25 (3.11)	767.53 (2.62)	767.23 (1.84)	765.40 (4.03)	2.12 < 2.57	0.12	Acceptable
Weak positive	4 ± 2 °C	273.35 (3.70)	265.20 (0.62)	265.73 (4.82)	265.23 (0.82)	263.83 (1.07)	262.71 (3.01)	264.64 (0.67)	2.28 < 2.57	0.11	Acceptable
	25 ± 5 °C	273.35 (3.70)	263.45 (2.70)	265.63 (2.47)	264.50 (0.57)	262.97 (2.60)	262.00 (3.46)	262.53 (0.56)	2.53 < 2.57	0.08	Acceptable
Negative	4 ± 2 °C	0.24 (0.03)	0.28 (0.03)	0.26 (0.01)	0.26 (0.02)	0.25 (0.02)	0.24 (0.02)	0.25 (0.02)	-	-	-
	25 ± 5 °C	0.24 (0.03)	0.33 (0.02)	0.46 (0.09)	0.32 (0.03)	0.35 (0.02)	0.39 (0.02)	0.38 (0.03)	-	-	-

COI: cutoff index; SD: standard deviation; QC: quality control. COI values represent the mean (SD) from triplicate measurements. Long-term stability was assessed over 6 months at 2–8 and 25 °C. For each QC level, independent two-sample *t*-tests showed no statistically significant differences (*p* > 0.05) between the two storage conditions.

## Data Availability

The dataset is available on request from the authors.

## Abbreviations

The following abbreviations are used in this manuscript:

RDT	Rapid diagnostic tests
HIV	Human immunodeficiency virus
IQC	Internal quality control
EQA	external quality assessment
Stabilizer	StabilZyme™ SELECT Stabilizer
ISO	International Organization of Standards
DTS	Dried tube specimens
FFP	Fresh frozen plasma
